# Screening for Scientific Skills in Spanish-Speaking Occupational Therapists (HACTO-Screen): Study Protocol of a Cross-Sectional Survey

**DOI:** 10.3390/healthcare9020124

**Published:** 2021-01-26

**Authors:** Daniel Prieto-Botella, Paula Fernández-Pires, Desirée Valera-Gran, Miriam Hurtado-Pomares, Cristina Espinosa-Sempere, Alicia Sánchez-Pérez, Iris Juárez-Leal, Paula Peral-Gómez, Eva María Navarrete-Muñoz

**Affiliations:** 1 Department of Surgery and Pathology, Miguel Hernández University, 03550 Alicante, Spain; daniel.prieto@alu.umh.es (D.P.-B.); paula.fernandezp@umh.es (P.F.-P.); mhurtado@umh.es (M.H.-P.); c.espinosa@umh.es (C.E.-S.); alicia.sanchez@umh.es (A.S.-P.); ijuarez@umh.es (I.J.-L.); pperal@umh.es (P.P.-G.); enavarrete@umh.es (E.M.N.-M.); 2 Grupo de Investigación en Terapia Ocupacional (InTeO), Miguel Hernández University, 03550 Alicante, Spain

**Keywords:** evidence-based practice, study protocol, occupational therapy, scientific skills, research training, research career development, healthcare education programs

## Abstract

The acquisition of scientific competencies for the application of evidence-based practice (EBP) is considered an essential part of healthcare education programs in order to improve clinical effectiveness. An examination of scientific skills in occupational therapists may be helpful in understanding their current practice as well as being useful in providing a basis for applying suitable approaches to the development and implementation of EBP. Hence, this study was designed with a double main objective: (1) to describe the level of acquisition of scientific skills and academic achievement in Spanish-speaking occupational therapists; (2) to examine the factors associated with these skills. The screening for Scientific Skills in Occupational Therapists (HAbilidades Científicas en Terapeutas Ocupacionales), the HACTO-Screen, is an online cross-sectional survey divided into five sections: sociodemographic data, academic and professional background; assessment of scientific skills; research training and development needs; experience as a researcher and/or academic. A total sample of 1159 occupational therapists finally participated. Main associations will be analyzed using multiple linear and/or Poisson regression models with/without robust variance. Our findings will provide valuable insights on the research skills and associated factors in a large sample of Spanish-speaking occupational therapists. The results will also be helpful to enhance research training and research career development in occupational therapy in order to promote the use of EBP.

## 1. Introduction

Occupational therapists as healthcare professionals should implement evidence-based practice (EBP) in order to ensure high-quality healthcare delivery and patient safety [1]. The application of EBP allows healthcare professionals to make clinical decisions that are supported by the best available up-to-date clinical information, expert experiences, and patients’ preferences [2]. In this respect, there is an international recognition that EBP is a keystone of healthcare professional education; however, the reality is that there are wide discrepancies between “best EBP” and actual clinical care, suggesting that effective development and implementation of EBP remains a crucial and academically challenging issue [3] that must be overcome.

In recent years, although the role and position of EBP have gained importance among occupational therapists [4], the integration of research findings into practice still has not become a routine task in their clinical performance [5,6,7,8]. One of the most plausible explanations of why the use of EBP keeps being a significant challenge for occupational therapists lies in the fact that research evidence for most interventions is still scant [9]. Indeed, most occupational therapy research to date has been conducted adopting an interpretative approach that gives preference to understanding experiences and/or perspectives of individuals or specific groups rather than undertaking initiatives aimed at evaluating healthcare interventions [4,6,10,11,12]. Thus, in terms of clinical decision-making, occupational therapists are clearly at a disadvantage compared with other healthcare professionals, since EBP requires the use of the best available evidence and is preferably developed from high-quality scientific studies [13].

There is existing literature aimed at identifying the barriers to and/or enablers to implementing evidence-based knowledge in occupational therapy practice. In 2013, a scoping review including 69 studies [6] revealed several individual and organizational determining factors in applying and integrating research evidence in occupational therapy. With regard to individual factors, this study suggested that positive attitudes, research preferences, participation in research, knowledge and skills, and higher confidence were important aspects to promote the use of research. Moreover, organizational factors such as system-level support, positive attitude and readiness of leaders and employers, available resources, and university support and partnerships were also identified as enablers of EBP. Using the criteria proposed by the Consolidated Framework for Implementation Research (CFIR) [14], a more recent systematic review based on 22 studies indicated several determinants that may influence how occupational therapists implement evidence-based knowledge in their practice [15]. In line with previously reported factors [6], it was suggested that adaptability of the practice, learning climate, leadership engagement, available resources, knowledge and beliefs about the intervention, individual stage of change, and executing the knowledge implementation strategy should be considered as relevant aspects when developing strategies for implementing EBP in occupational therapy [15]. Nevertheless, understanding the process of EBP in occupational therapy also includes, in parallel, identifying barriers to its use. Intriguingly, despite the available literature suggesting that EBP is widely perceived as positive by occupational therapists, several studies identified the lack of confidence and skills in appraising research analysis as a common obstacle to applying research-based knowledge in occupational therapy practice [4,6,8,16,17,18]. Moreover, some studies also indicated that limited time available, restrictions on access to research literature, fieldwork educators not practicing EBP, and/or putting higher value on clinical experience rather than research were seen as barriers particularly relevant to clinical settings or the workplace [4,6,7,16,17,18,19,20].

In the light of the circumstances, it must be noted that scientific skills constitute a key element in the EBP development as well as a crucial first step to implement EBP strategies [2,3]. Importantly, the acquisition of scientific competencies for the application of EBP should be integrated as an essential part into healthcare professional education programs to improve clinical effectiveness. In this sense, given that higher academic level was identified as a factor supporting the use of research [6], academic achievement could be seen as a good proxy for assessing EBP competence. From the perspective of occupational therapists, examining scientific skills could significantly contribute to a clearer understanding of the current trends in occupational therapy practice as well as be useful in providing a basis for applying suitable approaches to the development and implementation of EBP in this healthcare discipline. As far as we know, no previous studies have described scientific skills and academic achievement in Spanish-speaking occupational therapists. Therefore, this study was designed with a double main objective: (1) to describe the level of acquisition of scientific skills and academic achievement in Spanish-speaking occupational therapists, and (2) to explore the factors associated to these scientific skills. 

## 2. Materials and Methods 

### 2.1. Study Design and Participants

The screening for Scientific Skills in Occupational Therapists (HAbilidades Científicas en Terapeutas Ocupacionales), the HACTO-Screen, is an online cross-sectional survey specifically aimed at assessing the level of scientific skills in order to identify lack of knowledge and confidence in research competence in Spanish-speaking occupational therapists. This survey forms part of a larger program of research, the HACTO (HAbilidades Científicas en Terapeutas Ocupacionales (Scientific Skills in Occupational Therapists)) project. Further information about this project is available at www.hacto.edu.umh.es. Potential participants of the HACTO-Screen were selected with a nonprobability convenience sampling method.

### 2.2. Procedure and Enrolment

The enrolment was carried out online from April to June 2020. Study participants were recruited using a campaign strategy divided into 3 periods: 1st period, from 15 to 19 April 2020; 2nd period, from 20 to 30 April 2020; 3rd period, from 1 to 15 May 2020. The recruitment campaign involved posting study invitations and advertisements on social networks such as Twitter, Linkedin, Facebook, Instagram, Telegram, and WhatsApp and emailing study information to education and professional organizations of occupational therapy from Spain and Spanish-speaking Latin American countries. To optimize this process, several informative materials such as infographics [21,22] and Youtube videos [23] were created in order to promote and deliver the study information. As a call to participation, the main purpose of these materials was to highlight the importance of the study for the development of occupational therapy practice. Other strategies to enhance participation included posting the response rates for each phase of the recruitment campaign. All participants were asked to respond to the study survey and to give their informed written consent. To maximize the participation rates, survey responses were accepted until 15 June, 2020. After reviewing all the information gathered, participants were excluded from the study if they did not have an occupational therapy degree (i.e., undergraduate students or other healthcare professionals) or did not provide informed written consent to participate. In this study, a total sample of 1159 participants were finally included.

### 2.3. Survey Instrument and Study Variables

The survey instrument was an ad hoc anonymous self-completion questionnaire designed using Google Forms according to the Checklist for Reporting Results of Internet E-Surveys (CHERRIES) [24]. The questionnaire consisted of 58 closed- and open-ended questions and took approximately 10–15 min to complete. To ensure that the questionnaire assessed what it was intended to assess, seven graduates in occupational therapy with research training completed and revised the questionnaire in order to detect likely grammatical and phrasing mistakes, and/or typos, as well as to make clear the premise of each item of the questionnaire. Based on their feedback, the questionnaire was further refined by adding some changes to provide a clearer organizational structure and to improve the understanding of the questions. The final version of questionnaire was divided into four sections to collect information of the participants: (1) personal data (i.e., sociodemographic, academic, and professional background); (2) assessment of scientific skills; (3) analysis of research training and development needs; (4) experience as a researcher and/or academic.

### 2.4. Main Outcome Measure: Scientific Skills

To evaluate scientific skills, we based the content on the Practice-Oriented Research Training (PORT) program specifically addressed to practicing physical and occupational therapists developed by Murphy et al. (2010) [25]. We refined and reorganized the PORT 14 items used to test the level of research skills and created nine new items. Each item represents a different scientific/research skill and is rated on a scale ranging from 1 (need further basic instruction) to 5 (able to perform independently and show improvement-seeking motivation). Unlike the original scoring that ranged from 1 (need further basic instruction) to 4 (able to perform independently), we valued positively the fact that a participant declared a clear intention to improve his/her evidence-based knowledge and research skills, thereby increasing the scale up to a five-point score. The total score and the score on each item can be obtained by summing up the respective values of the items. Higher scores imply a better research performance, and the total maximum score that can be obtained is 115 points. Table 1 displays the general description of the research skills included in the HACTO-Screen survey.

### 2.5. Secondary Outcome Measure: Research Experience and Academic Achievement

Since the acquisition of scientific skills should be intrinsically linked to a research and/or academic career, a set of questions was specifically designed to evaluate the experience acquired as a researcher and/or academic. A summary of the information regarding research or academic experience data is presented in Table 2.

### 2.6. Other Outcome Measures

#### 2.6.1. Research Training and Development Needs

We developed a set of specific questions to identify training and development needs for research performance (Table 3). The information collected will be used to design training resources and support materials specifically aimed at developing research competence.

#### 2.6.2. General Characteristics and Sociodemographic Data

Basic information about sociodemographic features such as country, region (only for Spanish participants), sex, working status and working hours per week, and age of participants was collected. Data on academic training information are displayed in Table 4.

### 2.7. Statistical Analysis

#### 2.7.1. Sample Size

Previous studies indicated that around 50%–70% of occupational therapists reported having difficulties in applying research skills [16,18,20,26,27,28]. In this study, to calculate the sample size, we used the following assumptions: a prevalence rate of low level of research skills at 65%, a margin of error of 3%, a significance level of 5%, a power of 80%, and a two-sided test, thereby obtaining a sample of 971 participants as optimal. The software R, version 4.0.2 (R Core Team. R: A language and environment for statistical computing. R Foundation for Statistical Computing (Vienna, Austria; http://www.r-project.org) was used to perform all the statistical procedures.

#### 2.7.2. Data Analysis Plan

Descriptive analyses will be estimated to obtain a general description of the participant characteristics: sociodemographic features and academic training information; level of scientific skills, training and development needs, as well as details of research and/or academic experience. Values for normally distributed continuous variables will be expressed as mean and standard deviation or as median and interquartile range for non-normally distributed continuous variables. We will use the Kolmogorov–Smirnov test to check the normal distribution of the continuous variables. Categorical variables will be presented as frequencies and percentages. 

To assess the relationship between scientific skills scores and study covariates, we will use bivariate regression models. All the significant covariates (*p* < 0.20) will be identified as potential confounders and used to build the core models. All the covariates associated with the scientific skills scores will be included at a level of *p* < 0.10 following a backward elimination procedure. Notwithstanding their statistical significance, these variables will be kept in the models if they change the magnitude of the main effects by more than 10%. 

Multiple linear and/or Poisson regression models with or without robust variance will be used to examine the association between scientific skills scores and the study covariates of interest. Moreover, to assess the robustness of the main findings, we will also perform sensitivity analyses. Statistical analyses will be conducted using software R, version 4.0.2, and all statistical tests will be bilateral, assuming a significance level of 5%.

### 2.8. Ethical Approval, Ethical Considerations and Dissemination

This study protocol received the ethical approval from the Research Compliance Office of the Miguel Hernández University (Expte.2020/2618). The research was carried out in accordance with the Declaration of Helsinki.

Before the enrolment in the study, all the participants were informed and received general information about the project and contact details of the person responsible for the project (E.M.N.-M.) in writing. As indicated in the details of the project, their participation in the study was voluntary. Prior to completing the survey, all participants provided an online informed consent agreement, and no incentive was offered to take part in this study. All the information collected by questionnaire was anonymized, and data confidentiality is warranted during the whole research process (i.e., data collection, data cleaning and dissemination of research results). The participants will be informed on the progress of the study. Findings from this study will be shared on social networks and the project website (www.hacto.edu.umh.es). Moreover, we also expect that research findings will be presented at international meetings and will be published in open access peer reviewed journals.

## 3. Discussion

It is widely recognized that research competence forms the basis for developing and implementing EBP [2,3]. Thus, assessing scientific skills in healthcare professionals has become absolutely imperative to assure the quality of care provided to patients. In response to this concern, the present survey has been undertaken to determine the current status of scientific skills in Spanish-speaking occupational therapists and their research/academic achievements, as well as to elucidate the factors that may be associated with these skills. Moreover, this study also intends to identify specific research training and development needs with the further purpose of bridging the gap of research knowledge and skills in the current occupational therapy practice. As far as we know, this is the first time that a large-scale cross-sectional study evaluates the level of scientific skills in a sample of Spanish-speaking occupational therapists. From an academic and clinical training perspective, the results of this study will yield useful information to map scientific/research literacy of these healthcare professionals and to build a proper research skill development framework for occupational therapy.

Ideally, healthcare education programs should produce competent graduates who care for and about patients, are technically proficient, keep fully up to date with knowledge and skills, and use suitable and reliable evidence to their practice [29]. Despite broad acceptance of the view that knowledge and skills for research learning and EBP should be the basis for healthcare education programs [2,3] including education programs for occupational therapy [30,31,32] the reality is that the development of research competence needed for EBP is poorly integrated into healthcare programs’ curriculum design [2,3]. According to the latest reports from international organizations such as the World Federation of Occupational Therapists or European Network of Occupational Therapy in Higher Education [31,32,33], there are evident weaknesses in the development of research skills that act as significant limiting factors behind the current condition of occupational therapy practice. Moreover, intrinsically linked to the lack of effective research training, there is a growing concern about the need for research career development in the occupational therapy discipline because of the low number of highly qualified graduates and well-trained researchers to date [31,32]. Hence, in line with the future strategic actions intended to guarantee ongoing progress of quality of professional education and EBP in occupational therapy [31,32,33], our study intends to serve a research tool with a dual function as both assessment and planning tools. As an assessment tool, in accordance with primary and secondary outcomes, the results of this study will help to provide a detailed description of research capacity in a large sample of Spanish-speaking occupational therapists from Spain and Latin America, to quantify how many of them are doing scientific research, as well as to characterize the level and quality of their research output. As a planning tool, the information relating to research training and development needs collected from the study participants will be particularly useful in designing specific research training and development programs and resources tailored to the needs of Spanish-speaking occupational therapists from Spain and Latin America. In this regard, following the path opened by the Institute of Medicine to improve patient care and wellbeing by making clinical decisions based on EBP [34], this study endorses several initiatives aimed at developing a competency framework for education in EBP and clinical effectiveness have been proposed for healthcare professional programs [2,29,35]. As such, we intend to integrate this approach into an occupational therapy education program using the findings that will be obtained from the present study.

Nevertheless, this study has several limitations that should be acknowledged. Firstly, all the data gathered from the study participants were self-reported, suggesting that a misclassification cannot be discarded. However, in the case of any potential inaccuracy in reporting, it should be considered as nondifferential. Moreover, although the survey instrument consisted of an ad hoc online questionnaire, the part of questionnaire used to assess the main outcome (i.e., scientific skills) was based on reliable and valid items from a training research program developed by the Michigan Institute for Clinical & Health Research (MICHR) and specifically addressed to occupational therapists [25]. To ensure the validity of this online survey and control likely biases resulting from the nonrepresentative nature of the Internet population and the self-selection of participants (i.e., volunteer effect), we used the Checklist for Reporting Results of Internet E-Surveys (CHERRIES) [24] and estimated a study sample size to preserve the degree of representativeness. However, another important limitation is that the selection of study participants was for convenience using a snowball sampling, and this could affect the generalization of the results. Moreover, since Internet connection and technological devices were necessary to complete the online questionnaire, likely digital inequalities due to their physical Internet access and/or Internet skills cannot be disregarded, suggesting that the population of occupational therapists from Spain and Spanish-speaking Latin American countries can be partly misrepresented. Finally, a potential study limitation due to sociocultural differences among the occupational therapists from different Spanish-speaking countries included in this study should be also acknowledged.

## 4. Conclusions

Research or scientific skills are considered to play a crucial role in the development of positive attitudes towards EBP skills and ability to apply EBP. As a key element in the clinical decision-making process based on the best available evidence, the application of EBP is absolutely essential to ensure high-quality healthcare delivery and patient safety. According to the latest reports on the current state and future trends in occupational therapy addressed to both professional and academic sectors [31,32], the apparent lack of progress in implementing EBP in professional education programs is causing serious concerns that call attention to the need to improve research training and research career development in occupational therapy. In this study, we propose an objective and reliable methodology to describe and determine research skills and associated factors in a large sample of Spanish-speaking occupational therapists from Spain and Latin America. Moreover, the assessment of training and development needs will provide important insights into the difficulties the occupational therapists face when applying EBP. In line with recent initiatives aimed at developing a competency framework for education in EBP and clinical effectiveness for healthcare professional programs [2,3,34], the findings of this study will be also used for purposes of enhancing research training and research career development in occupational therapy discipline. Finally, we hope that our findings will constitute a suitable rationale for replicating in further samples to promote the use of EBP in occupational therapy.

## Figures and Tables

**Table 1:** Summary of the items * to test research skills included in the HACTO-Screen survey.

**Item 1.**	Translate research evidence into practice.
**Item 2.**	Identify a question that a research study might fill.
**Item 3.**	Formulate a research question (using PICO format).
**Item 4.**	Perform a literature search using an academic research database.
**Item 5.**	Write a testable hypothesis (based on my research question)
**Item 6.**	Choose the most suitable methodology to carry out a study.
**Item 7.**	Select the most suitable design for a study.
**Item 8.**	Select the measures to use in a study.
**Item 9.**	Estimate a sample size for a study.
**Item 10.**	Select a sample for a study.
**Item 11.**	Select the statistical analysis to test a study hypothesis.
**Item 12.**	Conduct the statistical analysis and interpret the results.
**Item 13.**	Present the findings from a research.
**Item 14.**	Write a grant to describe my study and argue that it merits funding.
**Item 15.**	Write a research paper.
**Item 16.**	Write a research paper published in a journal indexed in Journal Citation Reports (JCR).
**Item 17.**	Write an abstract for an international scientific congress.
**Item 18.**	Prepare a poster for a scientific congress.
**Item 19.**	Make an oral presentation at a scientific congress.
**Item 20.**	Write an abstract in English for an international scientific congress.
**Item 21.**	Prepare a poster in English for a scientific congress.
**Item 22.**	Appraise critically research studies for quality and applicability to practice.
**Item 23.**	Implement evidence-based knowledge in occupational therapy practice.

Abbreviations: HACTO-Screen, Screening for Scientific Skills in Spanish-Speaking Occupational Therapists; PICO, Population, Intervention, Comparison and Outcome; * Rating scale ranged from 1 to 5: 1 = need further basic instruction; 2 = able to perform with close supervision; 3 = able to perform with minimal supervision; 4 = able to perform independently; 5 = able to perform independently and show improvement-seeking motivation.

**Table 2:** Summary of the data on research or academic experience collected in the HACTO-Screen survey.

Do research during your working hours (yes; no)
Time dedicated to do research (hours per week)
Number of research papers published (total n)
Number of research papers published in scientific journals indexed in the JCR (total n)
Number of research papers published in scientific journals indexed in the 1st quartile of the JCR (total n)
Number of research papers published with leading authorship, i.e., first, last or corresponding authorship (total n)
Number of research projects with public or private funding in which you participated as principal investigator or co-investigator (total n)
Number of oral presentations on research findings during the present year (total n)
Have academic achievement according to research merits awarded by national agency *, i.e., “six-year term” (yes; no)
Number of “six-year terms” officially recognized (total n)
Number of research papers read during the last year (<20; 20–50; 50–100; >100)
Courses training completed during the last year (research methodology; statistical analysis; evidence-based occupational therapy; scientific literature search; epidemiology; scientific paper writing; presentation/dissemination of research findings; I did not take any course)

* In Spain, the CNEAI (Comisión Nacional Evaluadora de la Actividad Investigadora (National Commission for the Evaluation of Research Activity)) evaluates research activity of academics and awards a productivity supplement per each six-year term dedicated to research.

**Table 3:** Summary of the data on training and development needs collected in the HACTO-Screen survey.

**Areas in which Training and/or Development Is Needed:**
Statistical analysis Creation and management of databases Management of statistical analysis software Epidemiology Research study design Design of research study protocols Scientific communication Development of research projects Scientific journal publishing Research paper writing
**Support Materials to Improve Your Scientific Skills:**
Terms that you would like to include, if we develop a glossary of research terms Topics or aspects of your interest, if we create informative research materials as infographics Topics or aspects of your interest, if we develop a research skills handbook for occupational therapists Topics or aspects of your interest, if we design a research training course/workshop

**Table 4:** Summary of the data on academic information collected in the HACTO-Screen survey.

Occupational therapy degree (3-year bachelor’s degree; course of adaptation *; 4-year bachelor’s degree)
Name of institution where you obtained your bachelor’s degree
Year when you finished your bachelor’s degree
Have a master’s degree (yes; no)
Name of the master’s degree studied
Year when you finished your master’s degree
Have a doctoral degree (yes; no)
Year when you finished your doctoral dissertation
Work at university as an academic (partial time (associate lecturer); full time; no)
Name of the university where you work

* This one-year course is aimed to obtain the corresponding undergraduate degree adapted to the new European Higher Education Area (EHEA) requirements of the Bologna process.

## Data Availability

All the study data will be available to interested researchers upon request to Eva-María Navarrete-Muñoz, who is the responsible for the HACTO project. Requests will be reviewed by the research team and will require a data transfer agreement.
